# Early results of surgery for femoroacetabular impingement in patients with osteonecrosis of femoral head

**DOI:** 10.1051/sicotj/2018038

**Published:** 2018-11-06

**Authors:** Tarun Goyal

**Affiliations:** Department of Orthopaedics, All India Institute of Medical Sciences − Rishikesh, Virbhadra Marg, Rishikesh 209201 India

**Keywords:** Femoroacetabular impingement, Hip arthroscopy, Osteochondroplasty, Osteonecrosis, Surgical dislocation.

## Abstract

*Purpose*: Femoroacetabular impingement and its surgical treatment have not been described before in osteonecrosis of femoral head. We present here outcomes of 15 patients with femoroacetabular impingement secondary to osteonecrosis of femoral head. This results from partial collapse of femoral head, particularly in the anterosuperior region, secondary to osteonecrosis. With subsequent remodelling, periphery of the femoral head flattens and osteophytes form in this area. All these patients were managed with open/arthroscopic osteochondroplasty of femoral head.

*Methods*: These patients were symptomatic for hip impingement. Cam deformity was studied using computed tomography and magnetic resonance imaging. In six patients open osteochondroplasty was carried out using surgical hip dislocation. In nine patients arthroscopic femoral head osteochondroplasty was done. All the patients were followed up for hip pain (VAS), Harris hip score (HHS), Western Ontario and McMaster Universities Osteoarthritis Index (WOMAC), and hip range of motion.

*Results*: A statistically significant improvement in the VAS for pain, HHS, and WOMAC score was noted. Average HHS improved from 71.3 (SD, 13) to 89.7 (SD, 14.5), *p*-value 0.0079. Average WOMAC improved from 73.6 (SD, 15.4) to 92.4 (SD, 16), *p*-value 0.0154. Impingement test became negative in all the patients. A significant improvement in hip ROM was noted. There was no conversion to total hip arthroplasty. All patients could sit on the floor cross-legged and squat.

*Conclusion*: Some patients with partial collapse of femoral head due to osteonecrosis present chiefly with symptoms of femoroacetabular impingement. They should be identified as osteochondroplasty gives successful results in these patients.

Level of evidence − IV

## Introduction

Cam-type femoroacetabular impingement (FAI) is due to a femoral head−neck deformity. A number of causes have been identified for such cam-type deformity, such as developmental abnormality in shape of femoral head, sequelae of slipped capital femoral epiphysis (SCFE), Legg–Calv*é*–Perthes disease (LCPD), post-infectious sequelae, and post-traumatic remodelling. But in majority of cases of cam-type deformity in adults, no antecedent cause can be identified [1].

Osteonecrosis of the femoral head (ONFH) is a result of inadequate blood supply leading to death of the osteocytes. Uneventful healing of ONFH is uncommon and variable amount of collapse occurs depending upon involvement of the femoral head [2,3]. In adults remodelling potential of the femoral head is limited and collapse due to ONFH leads to irreversible changes in the shape of femoral head. Size and site of necrosis in the femoral head have been identified as reliable prognostic indicators of femoral head collapse [4–6]. But we have noticed that some patients with a large-size lesion in the weight-bearing area do not show extensive collapse of the femoral head. Contrary to the expectation, these patients do not progress to advanced degeneration of the hip joint. Rather they presented with FAI due to deformity at femur head–neck junction.

**Figure 1:**
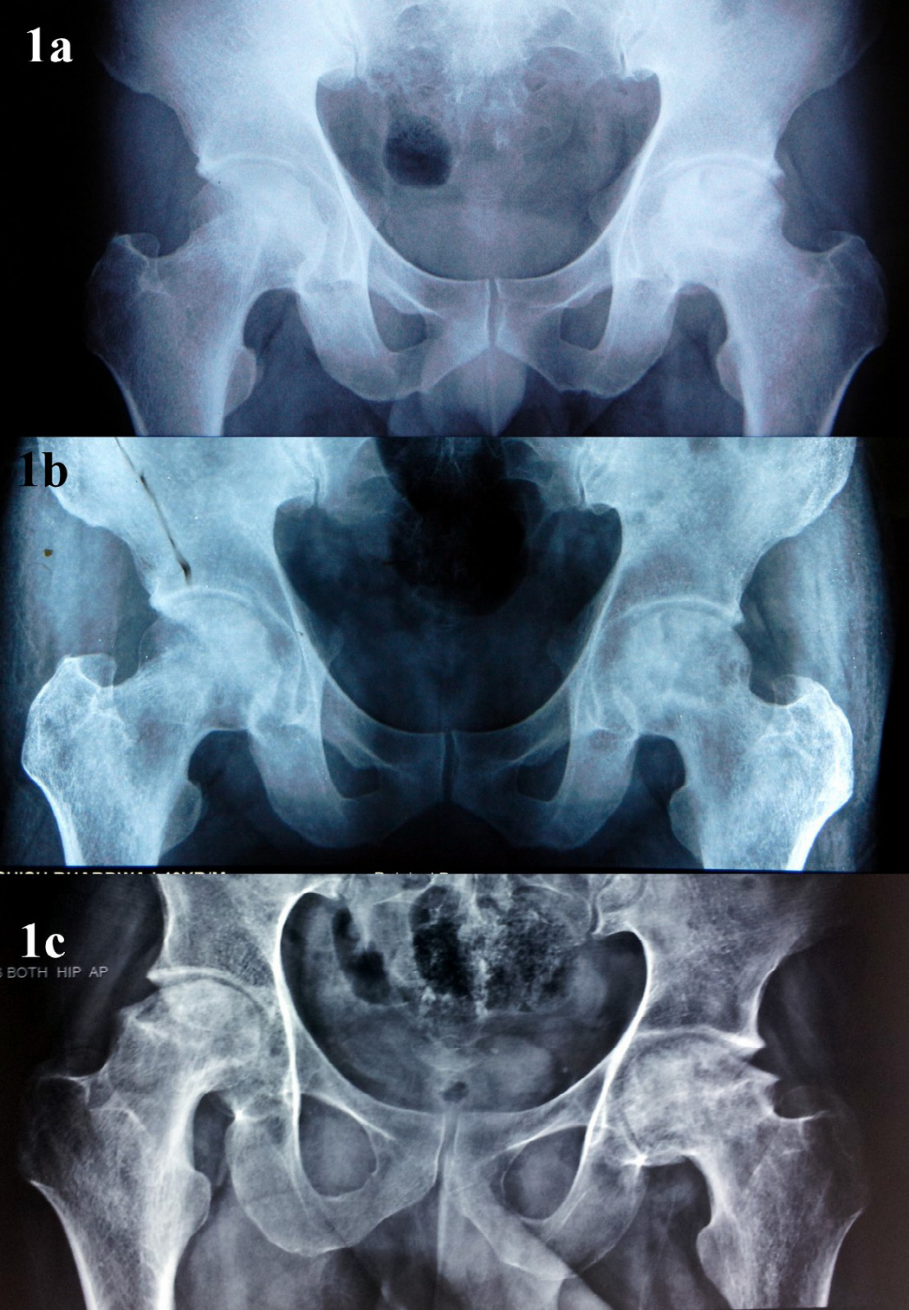
Serial radiographs of a 40 year old male with ONFH bilaterally: panel a: early stages showing density changes, more pronounced in left hip; panel b: after 3 months, fragmentation changes have appeared in both femoral heads. Femoral head collapse is eminent at this point; panel c: 1 year follow-up shows partial collapse in both femoral heads, relatively preserved joint space, relative flattening at the lateral periphery of the femoral heads, and formation of impinging cam-type lesion.

**Figure 2:**
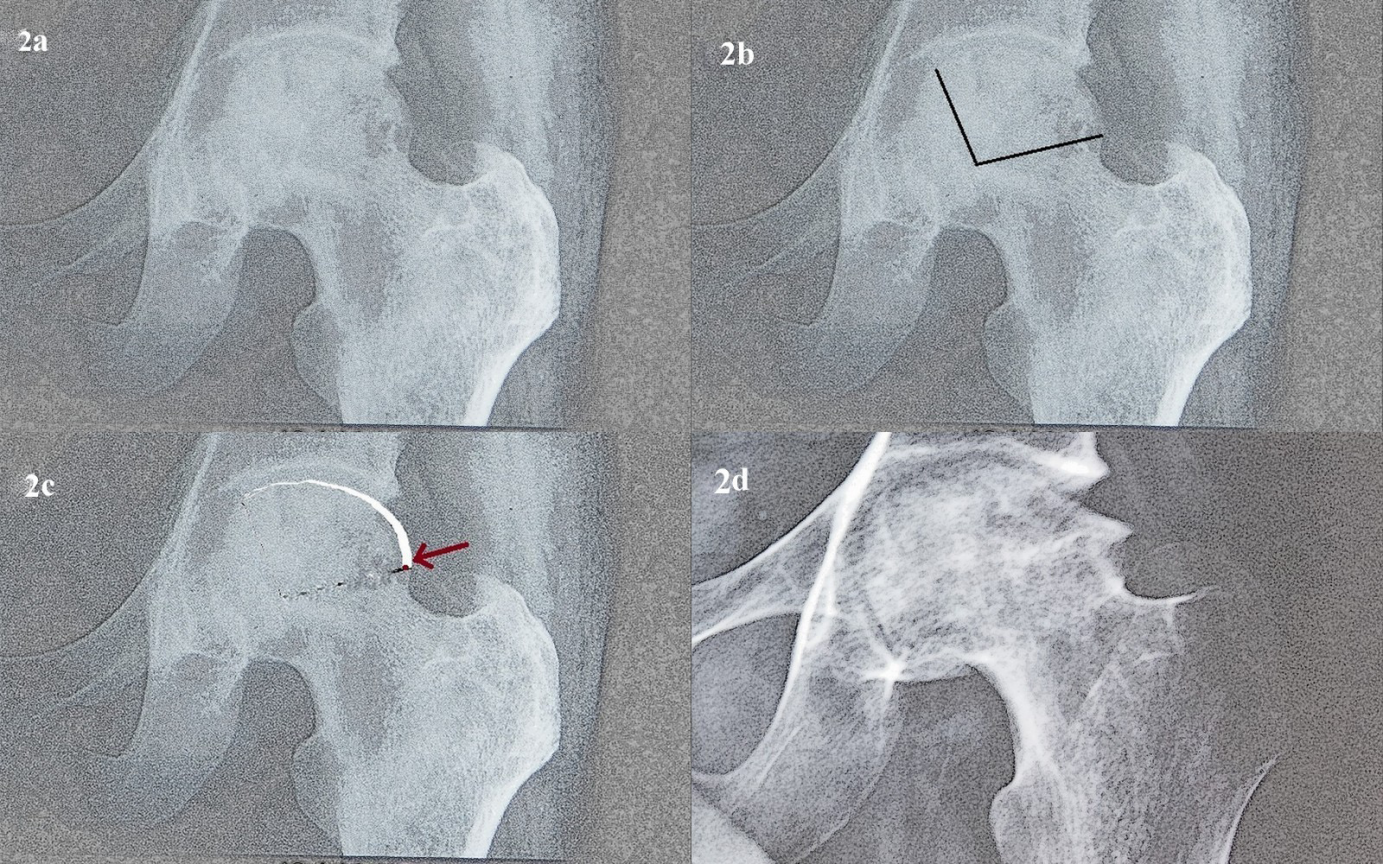
The mechanism of development of cam-type FAI in femoral heads after osteonecrosis: panel a: fragmentation in the femoral head with preservation of femoral head sphericity; panel b: it can be noticed from the radiograph that a large part of the femoral head is involved in the disease process; panel c: illustration of collapse of the femoral head. The entire diseased fragment undergoes collapse due to weakening of deeper metaphyseal bone. Subchondral bone and metaphyseal bone immediately deep to it remain intact; panel d: with remodelling of the step formed at anterolateral part of femoral head, a cam deformity develops.

FAI as a result of healed ONFH has not been described in literature before. We are describing this entity where partial collapse of femoral head occurs, particularly in the anterosuperior region, secondary to ONFH (Figure 1). A step deformity develops at the junction of this collapsed segment with normal bone in femur head–neck region (Figure 2). With subsequent remodelling, periphery of the femoral head flattens and osteophytes form in this area. We have encountered 15 such patients who presented to us with hip pain and classical signs and symptoms of FAI in whom we could confirm that the antecedent cause was ONFH using previous MRI scans. All these patients were managed with open or arthroscopic osteochondroplasty and follow-up results of these patients are presented in this article.

## Materials and methods

This case series included 15 patients (15 hips) who underwent surgery in our centre between January 2014 and January 2016. Clearance from the Institutional Ethics Committee was taken for this study. Informed consent was obtained from all the patients for inclusion in the study.

Inclusion criteria:

- patients with little or no hip pain on standing and walking, which did not interfere with routine activities or necessitated use of walking aid;
- patients symptomatic mostly on hip motion particularly flexion and hip rotations;
- near normal joint space on plain radiographs, or more than 50% joint space preserved laterally compared to opposite hip;
- MRI showing a continuous lining of cartilage on acetabular and femoral head;
- duration of symptoms more than 2 years to ensure that the hip has undergone sufficient remodelling and further collapse due to ONFH is not expected;
- presence of a cam-type lesion on femoral head–neck junction on radiographs/CT scans;
- presence of at least 50% of the normal arc of hip motion in coronal and sagittal planes with no fixed deformities.

In six patients open osteochondroplasty was carried out using surgical hip dislocation as described by Ganz et al. [7]. In nine patients arthroscopic femoral head osteochondroplasty was carried out. Technique used for hip was arthroscopy, which was similar to that described by Dienst et al. [8] where peripheral compartment arthroscopy is carried out first to address the femoral head–neck lesion. Indication for choosing open procedure over hip arthroscopy was more severe involvement of the femoral head with flattening of a larger circumference of the head–neck junction.

Preoperatively plain radiographs, magnetic resonance imaging (MRI), and computed tomography (CT) with 3D reconstruction were performed in all the patients for delineation of the lesion. Diagnosis of ONFH in all the patients was based on previous magnetic MRI records. Preoperative MRI scans of these patients were also evaluated for percentage area of femoral head involvement using method described by Kim et al. [9].

### Details of arthroscopic procedure

Hip arthroscopy was carried out in supine position on a fracture table with a perineal post. An unscrubbed assistant could manipulate the operated limb in positions of flexion/extension, rotations, and abduction/adduction on the limb of the fracture table. No traction was used for the procedure. If at all required, traction was used at the end of the osteochondroplasty procedure to visualise the articular cartilage. 1 mg/L of epinephrine was added to the irrigation fluid.

Standard anterolateral portal was used to insert the arthroscope. Image intensifier was used to confirm the position of the cannulated guide pin in the femoral head–neck area. A Nitinol wire was passed through the pin and the tract is dilated using dilators. Blunt trocar and a flow-sheath of arthroscope were introduced to feel bony contact in the anterior part of femoral neck to confirm its intra-articular placement. Arthroscope was then introduced. Alternatively, an outside-in capsulotomy could be carried out as described by Thaunat et al. [10]. Standard anterior portal was used for instrumentation. Attempt was made to identify the normal articular cartilage just peripheral to the labrum in neutral position of the limb. This would help to identify the extent of the deformed head–neck portion (cam deformity). Extension of the hip joint would help to identify the anterior cam deformity, and internal rotation helped to identify the lateral cam deformity.

It is important to understand that the goal of the surgery is mainly to remove only those part of the cam deformity which are causing impingement in flexion and rotations. In patients with more circumferential lesions, there is a possibility that some parts of this deformity located posteriorly (posterolateral and posteromedial) may be left after the procedure if they are not found to be contributing to impingement.

Hip range of motion was carried out perioperatively for dynamic assessment of femoroacetabular contact and this was also confirmed fluoroscopically. All the patients were followed up prospectively for hip pain (VAS for pain), Harris hip score (HHS), Western Ontario and McMaster Universities Osteoarthritis Index (WOMAC), hip movements in flexion, abduction, adduction, and arc of rotation. End point for success of this procedure was total hip replacement. Preoperative and post-operative functional outcome scores and range of motion were compared for statistical significance using Wilcoxon signed rank test.

Hip range of motion exercises were started as soon as the pain allowed, generally in first 1–2 days. Post-operative rehabilitation protocol included 6 weeks of protected weight bearing for patients undergoing surgical dislocation of hip. Immediate weight bearing was started for patients undergoing arthroscopic osteochondroplasty.

## Results

Mean age of the patients was 27.5 years (range 18–34 years). Mean duration of hip symptoms was 2.9 years (2.1–4.2 years). Hip joint space was normal or only slightly reduced in all the patients. Preoperative MRI scans of all these patients showed findings consistent with ONFH in all the patients. Average percentage area of femoral head involvement in these patients was 37% (range 28–50%).

At the time of surgery, articular cartilage in the weight-bearing area of the hip joint was found intact in all patients. Degenerative changes in the labrum at the site of impingement were seen in all the patients. Debridement of these tears was carried out. Labral repair was not required in any of these patients. A statistically significant improvement in the VAS for pain, HHS, and WOMAC score was noted in all the patients (Table 1). Average HHS improved from 71.3 (SD, 13) to 89.7 (SD, 14.5), *p*-value 0.0079. Average WOMAC improved from 73.6 (SD, 15.4) to 92.4 (SD, 16), *p*-value 0.0154.

**Table 1:** Preoperative and post-operative parameters of the patients in this series. There was a statistically significant difference noticed in all the parameters.

Parameter	Preoperative value	Post-operative value	*p*-value
VAS score in the position of impingement	5.13 (SD, 1.2)	1.6 (SD, 0.63)	0.00032
Harris hip score	71.3 (SD, 13)	89.7 (SD, 14.5)	0.0079
WOMAC	73.6 (SD, 15.4)	92.4 (SD, 16)	0.0154
Range of motion			
Flexion	92.1 (SD, 10.7)	120.47 (SD, 5.8)	0.0003
Internal rotation	15.6	21.9 (SD, 2.6)	0.0006
External rotation	13.2 (SD, 3.0)	28.9 (SD, 3.2)	0.0006
Adduction	17.1 (SD, 3.8)	28.0 (SD, 3.2)	0.0006
Abduction	19.5 (SD, 6.0)	39.3 (SD,4.0)	0.0003

VAS: visual analogue scale, WOMAC: Western Ontario and McMaster Universities Osteoarthritis Index, SD: standard deviation.

**Figure 3:**
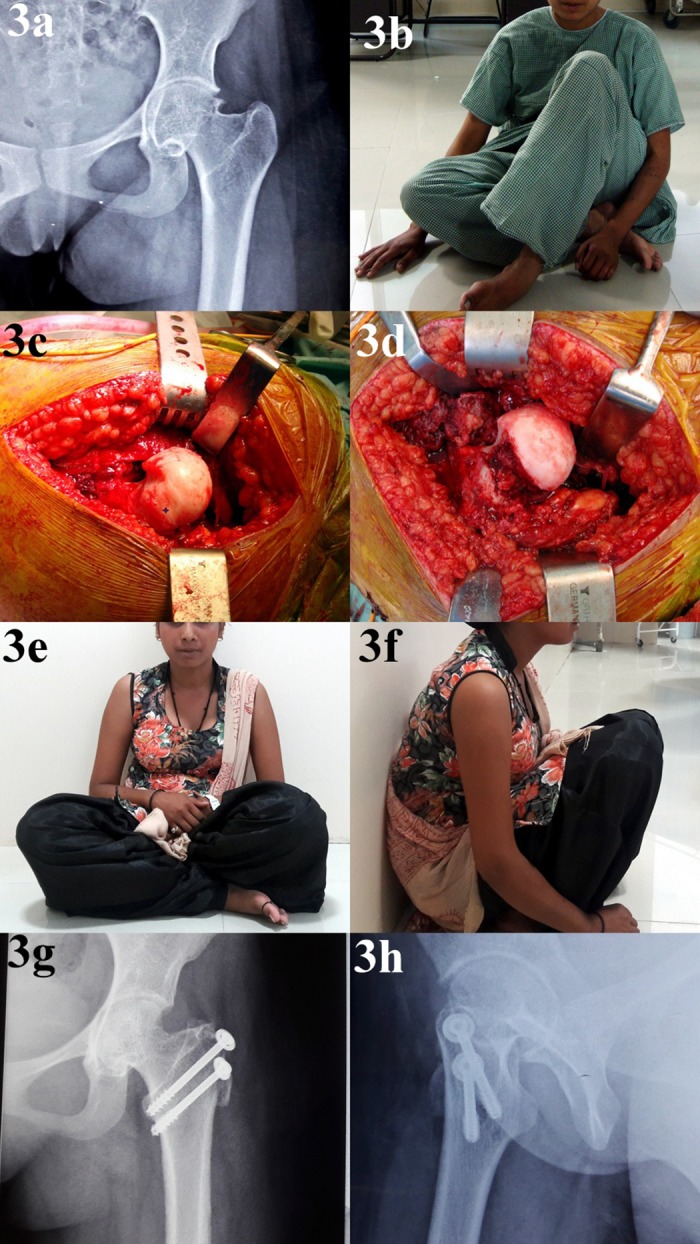
Details of a 27-year-old lady with idiopathic ONFH: panel a: preoperative radiograph showing cam deformity at femoral head–neck junction; panel b: she presented with painful restriction of flexion and internal rotation; panel c: intraoperative picture showing a large cam deformity. Femoral head cartilage looks normal; panel d: intraoperative image after femoral of cam deformity; panels e and f: follow-up pictures showing near normal hip range of motion. Floor-level activities such as squatting and sitting cross-legged are restored; panels g and h: post-operative radiograph showing normal appearing joint after removal of cam deformity.

**Figure 4:**
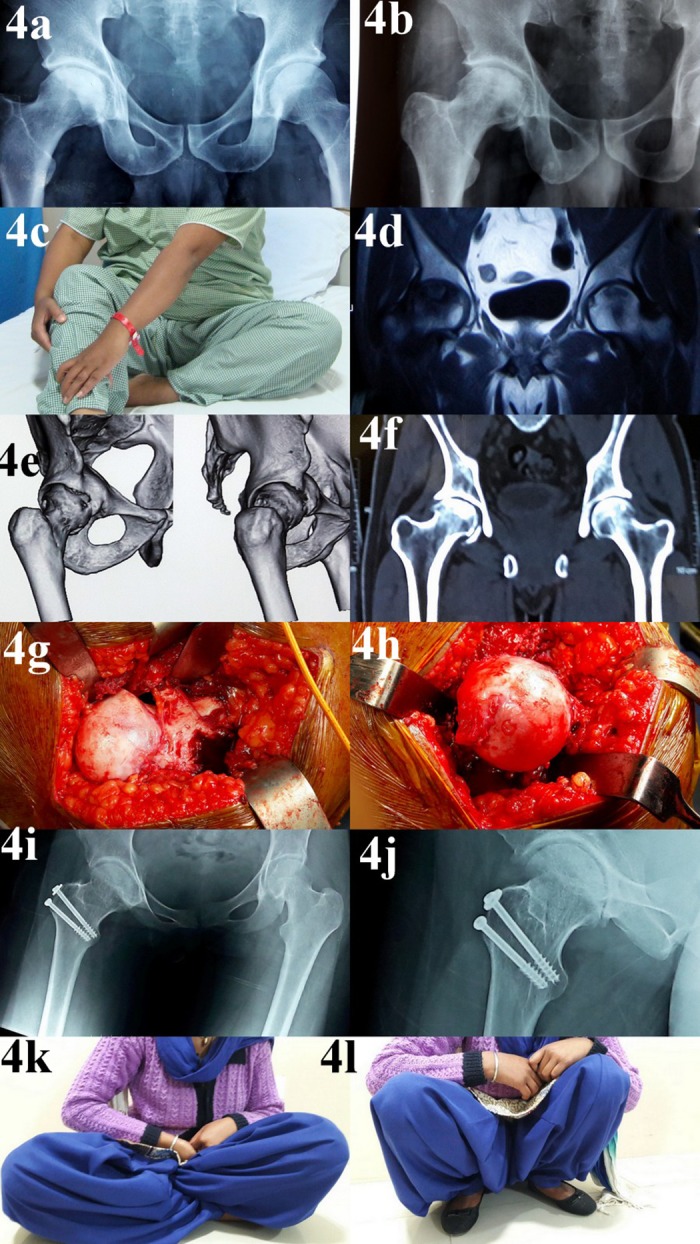
Follow-up of a 32-year-old female presenting with symptoms of hip impingement after idiopathic ONFH: panel a: plain radiograph early in the course of the disease showing partial collapse of femoral head; panel b: plain radiograph later in the course of disease showing cam deformity appearing due to remodelling; panel c: patients had symptoms of impingement during hip flexion and rotations; panel d: MRI scan early in the course of the disease-confirming changes of ONFH; panels e and f: CT scan of the same patient showing the cam deformity; panel g: intraoperative picture showing cam deformity at femoral head–neck junction; panel h: intraoperative picture after femoral head osteochondroplasty; panels i and j: post-operative radiographs showing restoration of femoral head sphericity; panels k and l: post-operative follow-up images showing restoration of hip range of motion.

**Figure 5:**
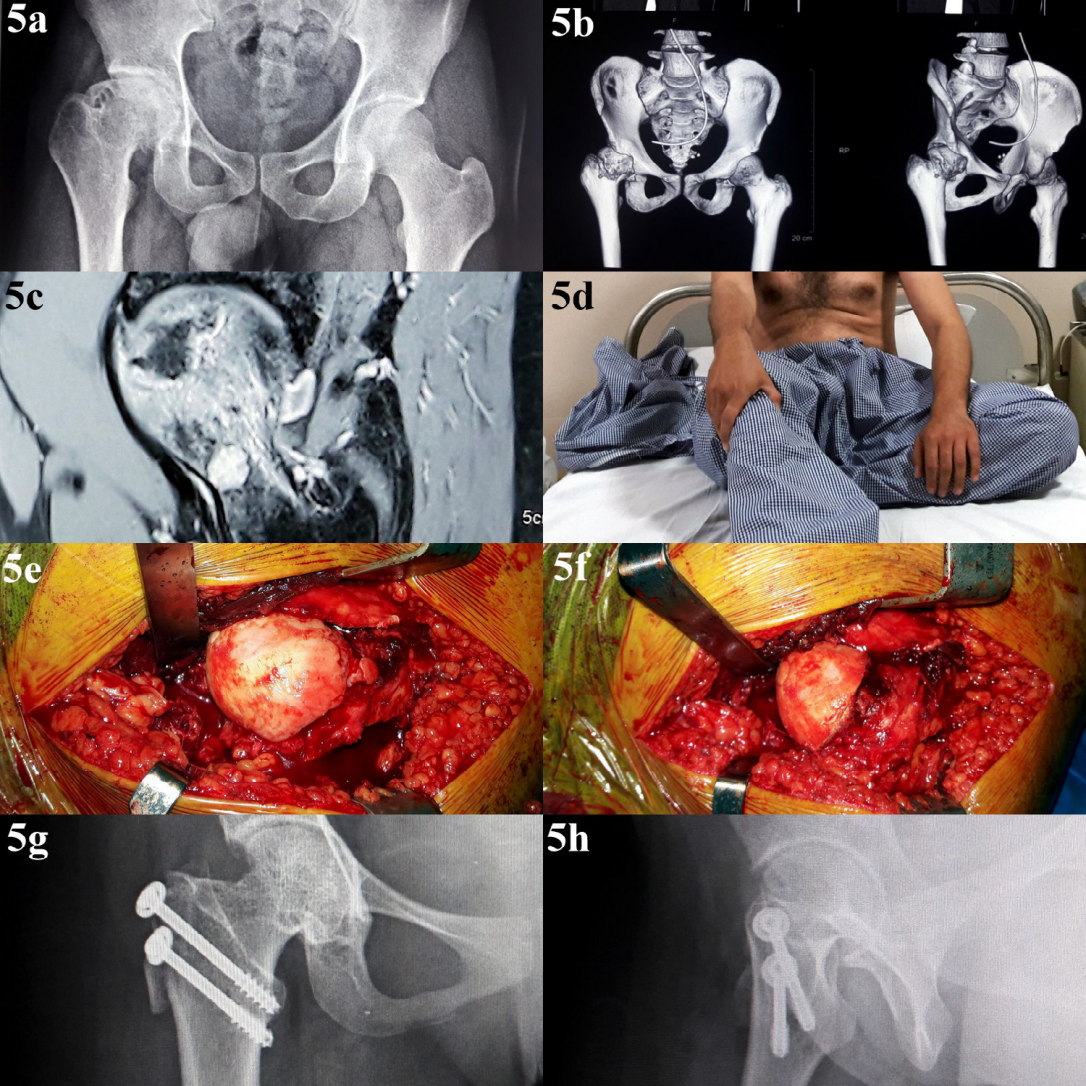
Follow-up of a 35-year-old male presenting with symptoms of hip impingement after steroid-induced ONFH; panel a: preoperative radiograph showing cam deformity in right femoral head; panel b: preoperative CT scan showing extent and location of cam deformity in the femoral head; panel c: MRI scan earlier in the course of disease-confirming changes of ONFH; panel d: clinical radiographs showing restricted hip motion due to cam deformity of femoral head; panel e: intraoperative picture showing cam deformity at femoral head–neck junction and preserved articular cartilage; panel f: intraoperative picture after femoral head osteochondroplasty; panels g and h: post-operative radiographs showing restoration of femoral head sphericity.

**Figure 6:**
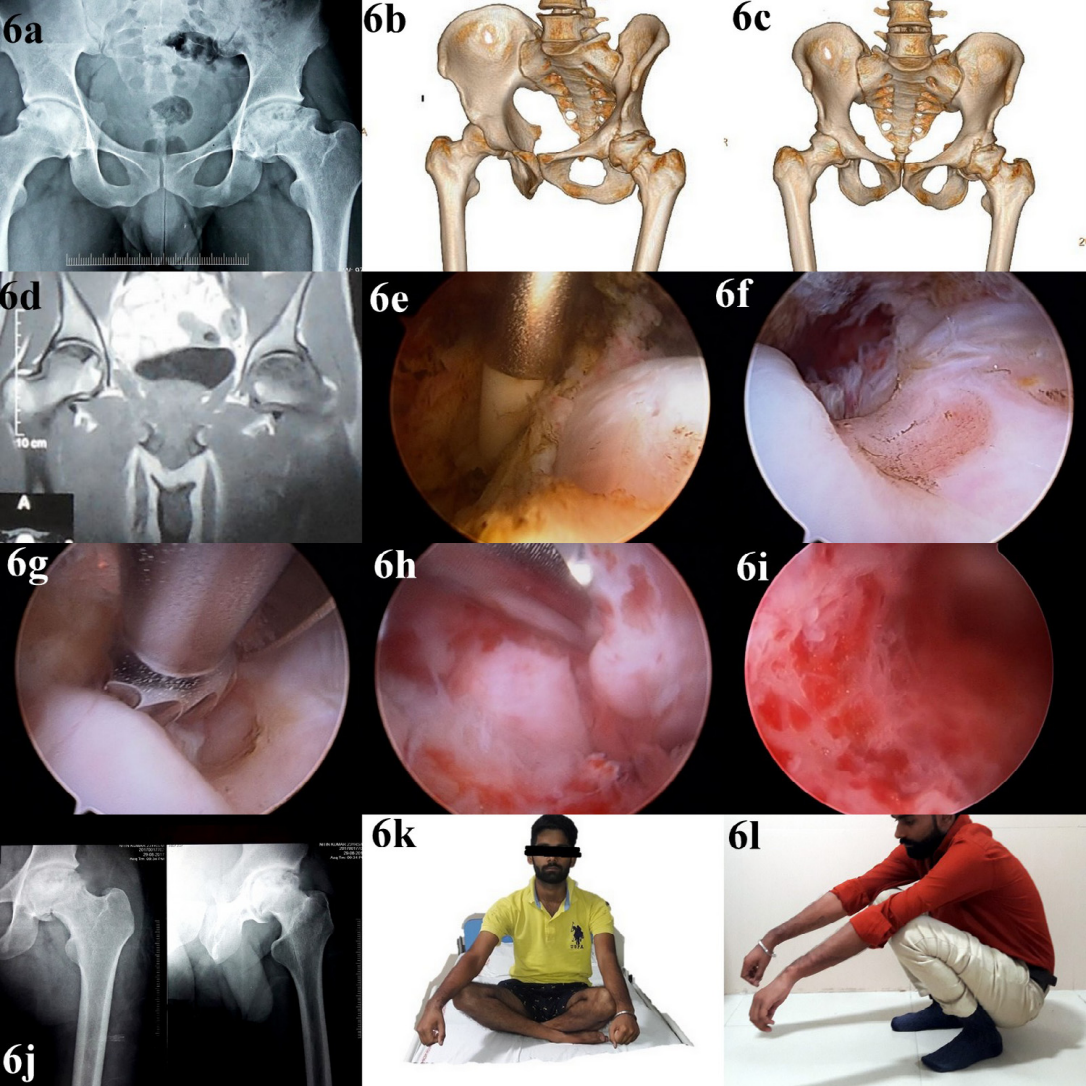
Follow-up of a 33-year-old male presenting with symptoms of hip impingement after alcohol-induced ONFH; panel a: preoperative radiograph showing cam deformity in right femoral head; panels b and c: preoperative CT scan showing extent and location of cam deformity in the femoral head; panel d: MRI of the hip joint done 1.8 years before the surgery confirming ONFH bilaterally; panel e: intraoperative image showing arthroscopy of the peripheral compartment of the hip joint using outside-in capsulotomy; panel f: intraoperative image of hip arthroscopy showing identification of the cam deformity; panel g: intraoperative image of hip arthroscopy showing resection of the cam deformity; panel h: intraoperative image of hip arthroscopy showing cam deformity in the anterolateral part of femoral neck; panel i: intraoperative image of hip arthroscopy after removal of the deformity; panel j: post-operative radiograph after removal of cam deformity; panels k and l: post-operative follow-up images showing restoration of hip range of motion.

**Figure 7:**
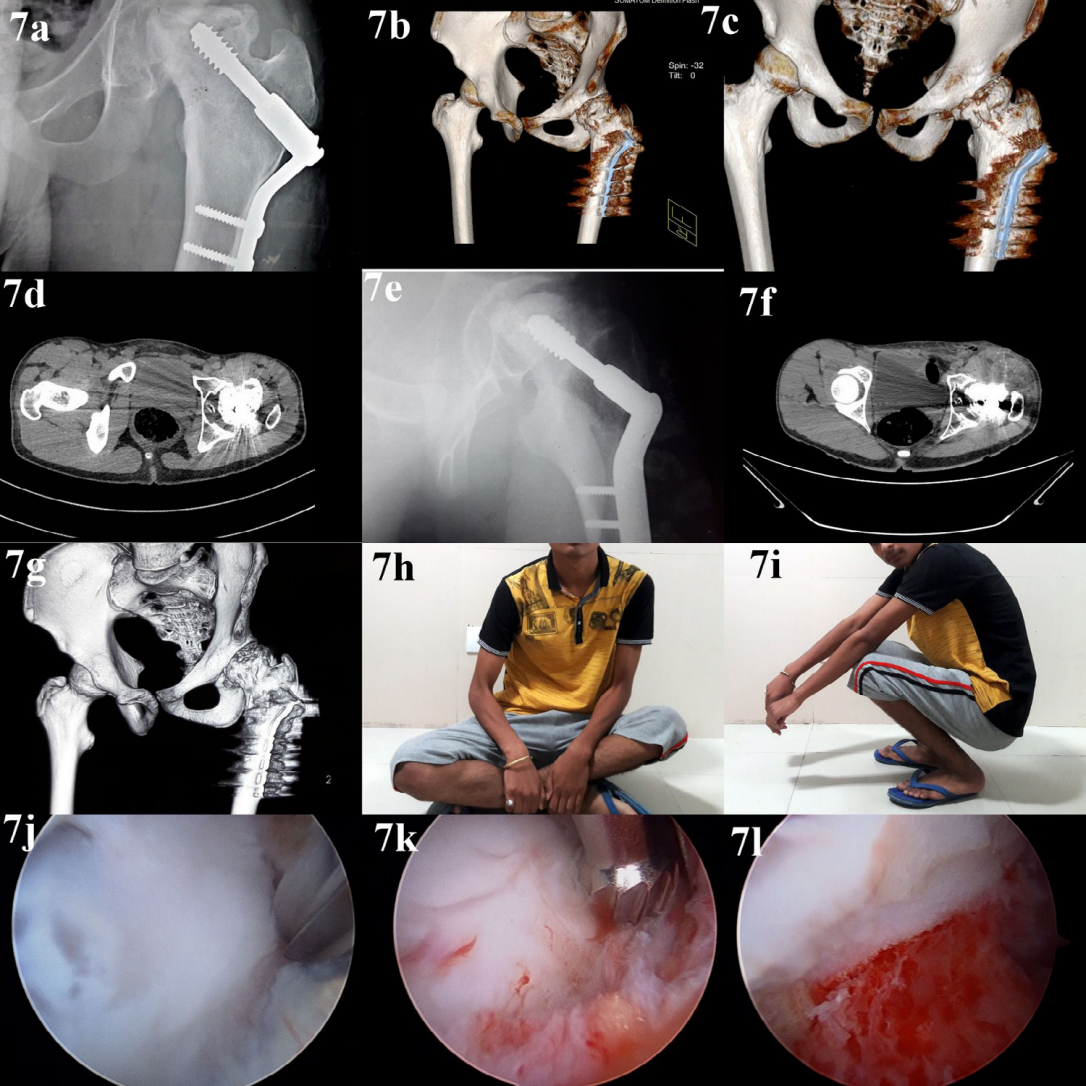
Follow-up of a 22-year-old male presenting with symptoms of hip impingement after post-traumatic ONFH. The patient initially sustained neck of femur fracture, which was treated with closed reduction and internal fixation with cannulated cancellous screws. The fracture failed to unite at 6 months and valgus osteotomy of proximal femur with internal fixation with a double-angle dynamic hip screw was carried out. The fracture united and patient was asymptomatic for 1 year. Subsequently, he presented with hip pain and radiograph showed collapse of femoral head indicating ONFH. He was managed conservatively for 1.5 years. He then developed symptoms of hip impingement, with restriction of hip flexion and rotations. There was no pain on weight bearing or walking. He was managed with arthroscopic osteochondroplasty: panel a: preoperative radiograph showing cam deformity in right femoral head; panels b–d: preoperative CT scan showing extent and location of cam deformity in the femoral head; panel e: post-operative radiograph showing restoration of femoral head sphericity; panels f and g: post-operative CT scan after removal of cam deformity; panels h and i: post-operative follow-up images showing restoration of hip range of motion; panel j: intraoperative image of hip arthroscopy showing identification of the cam deformity; panel k: intraoperative image of hip arthroscopy showing resection of the cam deformity; panel l: intraoperative image of hip arthroscopy after removal of the deformity.

Impingement test became negative in all the patients after the surgery. A significant improvement in hip ROM was noted (Table 1). There was no conversion to total hip arthroplasty at the final follow-up. All patients could sit on the floor cross-legged and squat. There were no instances of osteonecrosis, superficial or deep infection, implant failure, or non-union of the trochanteric osteotomy site after the surgery. Average follow-up duration was 2 years (range 1.5–2.5 years). Preoperative and post-operative follow-up of representative cases is shown in Figures 3–7.

## Discussion

We propose that the cause of anterosuperior femoral head–neck deformity is partial collapse of the femoral head due to osteonecrosis. It is not easy to understand why behaviour of these hips differed from the more usual event of advanced collapse in ONFH. ONFH most commonly involves the anterosuperolateral part of the femoral head. Involvement is generally in shape of a cone with base towards the articular surface. Biomechanically the cortical shell of the femoral head is stiffer than the underlying cancellous bone [10,11]. In ONFH the support of the cortical shell from underlying cancellous bone weakens due to necrosis of bone. If the cortical shell and underlying subchondral bone is strong, the entire conical area of the involved part of the head may depress as a single piece with buckling of the central metaphyseal bone. This collapse is generally small if good-quality bone is present in subchondral region. This anterosuperior part of the femoral head becomes relatively aspherical and a step appears at its junction with the normal bone laterally. With remodelling, peripheral osteophytes start appearing at this periphery.

In all our patients, a larger part of the femoral head was involved, including weight-bearing area and a large conical part of underlying femoral metaphysis, reaching up to the centre of the femoral head. This entire part sequestered together during collapse forming a step deformity at the periphery of the head or the head–neck junction. With revascularisation, repair of this lesion had occurred, as evidenced by this segment being well fixed and normally bleeding during the surgery.

Surgical options for hip preservation surgery in ONFH include core decompression or bone grafting in precollapse stage and redirectional osteotomies once collapse ensues. Hip arthroplasty is the standard treatment for hips involved with osteonecrosis. As these patients are young, most of the patients undergoing arthroplasty can expect more surgeries of their hip joints in future. Hip preservation should be attempted in these patients if possible. Osteochondroplasty of the femoral head–neck junction has not been described as an option for hip preservation in ONFH before. These patients with partial collapse of the head predominantly have symptoms of FAI. They have little pain while weight bearing and the sphericity of femoral head in weight-bearing region is relatively maintained. A significant pain relief and restoration of function can be expected in these patients after osteochondroplasty of the femoral head to remove the cam deformity and restoration of the femoral head–neck contour.

Natural history of ONFH is still incompletely understood. These findings also bring to our notice deficiencies in current understanding of changes occurring in the subchondral and metaphyseal bone in ONFH and mechanisms of head collapse and healing. One important concern in these patients with ONFH is possibility of progression of the collapse of the femoral head. All these patients were symptomatic for more than 2 years, which indicates that the vascular insult to the hip was old and remodelling has already occurred in the head. No further collapse was noted in any of these patients 2 years after osteochondroplasty. As these patients presented with a diagnosis of ONFH, presence of hip symptoms, and radiological changes in the femoral head, they had been advised hip arthroplasties before they were seen in our hospital.

## Conclusion

Some patients with partial collapse of femoral head due to osteonecrosis present chiefly with symptoms of femoroacetabular impingement. They should be identified as femoral head–neck osteochondroplasty gives successful results in these patients. This article adds a new dimension to femoral head preservation surgery in carefully selected patients with osteonecrosis of femoral head. This study presents only a short-term follow-up. A longer follow-up of these patients is awaited and should be available in future.

## Conflict of interest

The authors declare that they have no conflicts of interest in relation to this article.
